# Cyber-Creativity: A Decalogue of Research Challenges

**DOI:** 10.3390/jintelligence13080103

**Published:** 2025-08-13

**Authors:** Giovanni Emanuele Corazza, Sergio Agnoli, Ana Jorge Artigau, Ronald A. Beghetto, Nathalie Bonnardel, Irene Coletto, Angela Faiella, Katusha Gerardini, Kenneth Gilhooly, Vlad P. Glăveanu, Michael Hanchett Hanson, Hansika Kapoor, James C. Kaufman, Yoed N. Kenett, Anatoliy V. Kharkhurin, Simone Luchini, Margaret Mangion, Mario Mirabile, Felix-Kingsley Obialo, Connie Phelps, Roni Reiter-Palmon, Jeb S. Puryear, Eleonora Diletta Sarcinella, Min Tang, Giulia Maria Vavassori, Florent Vinchon, Indre Viskontas, Selina Weiss, Dimitrios Zbainos, Todd Lubart

**Affiliations:** 1 DEI Department, Marconi Institute for Creativity, University of Bologna, Viale Risorgimento 2, 40136 Bologna, Italy; irene.coletto@gmail.com (I.C.); angela.faiella@unibo.it (A.F.); mario.mirabile2@unibo.it (M.M.); giulia.f.vavassori@gmail.com (G.M.V.); 2 Department of Life Sciences, University of Trieste, Via E. Weiss, 2, 34128 Trieste, Italy; sergio.agnoli@units.it; 3 School of Communication, Universidad Austral, Mariano Acosta 1611, Pilar B1630FHB, Provincia de Buenos Aires, Argentina; ajorge@austral.edu.ar; 4 Mary Lou Fulton College for Teaching and Learning Innovation, Arizona State University, 1050 S. Forrest Mall, Tempe, AZ 85281, USA; ronald.beghetto@asu.edu; 5 InCIAM & PSYCLE, Aix-Marseille University, 29 Avenue Robert Schuman, 13621 Aix-en-Provence cedex 01, France; nathalie.bonnardel@univ-amu.fr; 6 Research Center in Communication Psychology (PsiCom), Department of Psychology, Catholic University of the Sacred Heart, Via Largo Fra Agostino Gemelli, 1, 25123 Milan, Italy; katusha.gerardini@unicatt.it (K.G.); eleonora.sarcinella1@unicatt.it (E.D.S.); 7 Psychology Department, University of Hertfordshire, College Lane, Hatfield AL10 9AB, UK; k.j.gilhooly@herts.ac.uk; 8 DCU Centre for Possibility Studies, Dublin City University, Glasnevin Campus, D09 V209 Dublin, Ireland; vlad.glaveanu@dcu.ie; 9 Centre for the Science of Learning and Technology, University of Bergen, Christiesgate 12, 5020 Bergen, Norway; 10 Department of Human Development, Teachers College, Columbia University, 525 W. 120th Street, New York, NY 10027, USA; mah59@tc.columbia.edu; 11 Institut de Psychologie, Université de Paris Cité, 71 Avenue Edouard Vaillant, 92774 Boulogne-Billancourt, France; 12 Department of Psychology, Monk Prayogshala, 4114, C Wing, Oberoi Garden Estates, Near Chandivali Studios, Powai, Mumbai 400072, India; hk@monkprayogshala.in; 13 Neag School of Education, University of Connecticut, 2131 Hillside Road, Unit 3007, Storrs, CT 06269, USA; james.kaufman@uconn.edu; 14 Faculty of Data and Decision Sciences, Technion—Israel Institute of Technology, Kiryat Hatechnion, Haifa 3200003, Israel; yoedk@technion.ac.il; 15 School of Psychology, HSE University, 20 Myasnitskaya Street, Moscow 101000, Russia; tovyharhur@gmail.com; 16 Department of Psychology, Pennsylvania State University, Moore Building, Fischer Road, State College, PA 16801, USA; simoneluchini@psu.edu; 17 The Edward de Bono Institute for Creative Thinking and Innovation, University of Malta, MSD2080 Msida, Malta; margaret.mangion@um.edu.mt; 18 Research Center on Intelligent Technologies (CITIUS), University of Santiago de Compostela (USC), Rúa de Jenaro de la Fuente Domínguez, 15782 Santiago de Compostela, Spain; 19 Centre for Creativity and Entrepreneurial Studies, Dominican University Ibadan, 10, Education Layout, Ibadan 200132, Nigeria; obialo.f@dui.edu.ng; 20 The Teachers College, Emporia State University, 1 Kellogg Place, Emporia, KS 66801, USA; cphelps@emporia.edu; 21 Department of Psychology, University of Nebraska at Omaha, 6001 Dodge St., Omaha, NE 68132, USA; rreiter-palmon@unomaha.edu; 22 Department of Teaching and Learning, University of Montana, 32 Campus Drive, Missoula, MT 59812, USA; jeb.puryear@umontana.edu; 23 University Institute of Schaffhausen, Rheinstrasse 10, CH-8200 Schaffhausen, Switzerland; min.tang@hochschule-schaffhausen.ch; 24 LaPEA, Université Paris Cité and Univ Gustave Eiffel, 71 Avenue Edouard Vaillant, 92100 Boulogne Billancourt, France; florent.vinchon@gmail.com (F.V.); todd.lubart@u-paris.fr (T.L.); 25 Department of Psychology, University of San Francisco, 2130 Fulton Ave., San Francisco, CA 94117, USA; ivviskontas@usfca.edu; 26 Department of Psychology, University of Hildesheim, Universitätsplatz 1, 31141 Hildesheim, Germany; selina.weiss@uni-hildesheim.de; 27 Department of Economics and Sustainable Development, Harokopio University, El. Veinzelou 70, 17676 Athens, Greece; zbainos@hua.gr

**Keywords:** creativity, cyber-creativity, artificial intelligence, AI, ethics, creative process, sociocultural creativity, education

## Abstract

Creativity is the primary driver of our cultural evolution. The astonishing potential of artificial intelligence (AI) and its possible application in the creative process poses an urgent and dramatic challenge for humanity; how can we maximize the benefits of AI while minimizing the associated risks? In this article, we identify all forms of human–AI collaboration in this realm as cyber-creativity. We introduce the following two forward-looking scenarios: a utopian vision for cyber-creativity, in which AI serves to enhance and not replace human creativity, and a dystopian view associated with the pre-emption of all human creative agency caused by the rise of AI. In our view, the scientific community is called to bring its contribution, however small, to help humanity make steps towards the utopian scenario, while avoiding the dystopian one. Here, we present a decalogue of research challenges identified for this purpose, touching upon the following dimensions: (1) the theoretical framework for cyber-creativity; (2) sociocultural perspectives; (3) the cyber-creative process; (4) the creative agent; (5) the co-creative team; (6) cyber-creative products; (7) cyber-creative domains; (8) cyber-creative education; (9) ethical aspects; and (10) the dark side of cyber-creativity. For each dimension, a brief review of the state-of-the-art is provided, followed by the identification of a main research challenge, then specified into a list of research questions. Whereas there is no claim that this decalogue of research challenges represents an exhaustive classification, which would be an impossible objective, it still should serve as a valid starting point for future (but urgent) research endeavors, with the ambition to provide a significant contribution to the understanding, development, and alignment of AI to human values the realm of creativity.

## 1. Introduction

Since the first industrial revolution, modern societies have been evolving at an increasing pace, with waves of change fundamentally driven by technological progress. In general, new technologies have the potential to enhance productivity and efficiency, and positivistic views tend to focus exclusively on the positive sides, neglecting any early concern for possible detrimental side effects. In fact, the technological positivist sees those who advocate caution as reactionary conservatives, a hurdle to be overcome. Throughout history, any major technological advancement has been confronted with negative social reactions, typically heralded by those who felt furthest away from the control and exploitation of the technology itself (Jones 2013).

In the Information Society, two trends emerge, personalization and distribution (Corazza et al. 2010). More and more, products, services, and experiences are tailored to individual needs and profiles, a characteristic enabled by the flexibility afforded by digital technologies. This leads to higher and higher levels of personalization, as well as to the construction of information bubbles (Spohr 2017), in which an individual tends to be trapped by a sort of confirmation cage. On the other hand, distributed information technologies have broken the rigid time–spatial structure of the Industrial Society, allowing groups to live and work together irrespective of their physical location. In a society in which technology allows everyone to have access to all information, and thus knowledge loses its differentiator trait, human dignity tends to be tied to the generation of original ideas; creativity is a democratic necessity in the Information Society (Corazza 2017).

We are now witnessing a transition towards the Post-Information Society, based on the contemporaneous surge of the following three technological waves: artificial intelligence (AI), super-computing, and the Internet of Things (IoT). None of these technologies have come as a surprise, given that their development has been ongoing for several decades (Brynjolfsson and McAfee 2014). Therefore, the question of defining the human role in a cyber–human collaboration has been on the table for quite some time. The expectation was that machines would take on the heavier and lower-level duties, while humans would elevate their activity and be ever more engaged in creative processes, to the point where creativity would have to be considered a basic necessity for meaningful survival (Corazza 2016, 2017).

However, the coming of age of computational creativity (Kantosalo and Takala 2020), and more recently of Generative AI (Gen-AI) (Maltoni et al. 2025) based on Artificial Neural Networks (ANN), signaled by the public appearance of ChatGPT in November 2022, has changed the situation in a dramatic way. Large Language Models (LLMs), trained on tera-bytes of data scraped from the Internet, have demonstrated capacities related to text “understanding” and production that surpassed everyone’s expectations, including those of the authors of the LLM algorithms themselves. This success in the domain of writing has quickly been matched by similar achievements in the production of image, voice, music, and video content, to the point that today LLM multimodality is an accepted feature. In parallel to these developments, AI algorithms have been developed to tackle widespread scientific challenges, such as protein folding rules, design of new materials, elaboration of astrophysical data, to name but a few examples. In a nutshell, in the Post-Information Society, we are dealing with the rapid emergence of AI, adding particular urgency to the need of finding answers to questions that have been on the table for a few decades. Surprisingly, Gen-AI has demonstrated creative capacities that apparently nearly match the level of professional human creatives, albeit under the conditions that a human has invested in prompting the LLM algorithm. At the time of writing, Gen-AI has not reached any significant level of autonomy; although, this fact is bound to change in the future.

No one should be ready to concede that creativity will ever become a sole domain for AI; for this reason, the term *cyber-creativity* is here introduced as an umbrella to cover the collaboration in creative tasks between one or more human agents and one or more artificially intelligent machines (Corazza 2025b). Note that in this definition the term “artificially intelligent machines” must be intended in the wide sense as any form of artificially intelligent algorithm implemented through isolated or networked systems. For our purposes, cyber-creativity should be intended in the widest possible sense, covering the entire spectrum of the creativity phenomenon in the Post-Information Society, characterized by a continuum in the degree of collaboration between humans and machines, from full autonomy of humans to full autonomy of machines, going through the indefinite number of intermediate levels in which humans and machines co-create with different levels of autonomy and modalities for collaboration.

Several authors agree that understanding cyber-creativity is a crucial and urgent challenge (Binz et al. 2023; Cropley and Cropley 2023; Glăveanu and de Saint-Laurent 2023; Marrone et al. 2024; Rafner et al. 2023; Vinchon et al. 2023). Exploring all dimensions of cyber-creativity, however, is far from being an easy task, in part because throughout its evolution humanity has held a firm belief that the creativity construct was its own distinctive playground, the very reason why our culture evolves exponentially (Enquist et al. 2008). However, this objective cannot be postponed, as AI is a very sharp double-edged sword; its huge potential for beneficial exploitation is accompanied by an equally astonishing menace of detrimental effects, up to the definitive existential risk for humanity (Bostrom 2014). Perhaps for the first time in history, it is necessary today to consider without delay both positive and negative scenarios for the evolution of cyber-creativity, in order to take actions that increase the potential for the former while mitigating the latter.

The purpose of this article is to help tackle the understanding and shaping of cyber-creativity in its multiple facets through a projection onto two antipodean alternative futures. Imagining living in the year 2035, we describe a utopian and a dystopian scenario for cyber-creativity, using a narrative style. Whereas there is no claim that these scenarios are accurate predictions of the future in any sense, they should suffice to provide the idea that AI’s impact on creativity could potentially be very positive or very negative, depending on humanity’s choices, decisions, and actions. Then, the guiding theme for developing our argument becomes that of identifying the main research challenges that should be pursued for “attracting” the utopian scenario and “avoiding” the dystopian scenario, in every identified dimension for scientific exploration. In total, we identified ten main research challenges and about sixty specific research questions. Note that the identification of research challenges and questions is well beyond the capacity of a single group of researchers; in this sense, in the description of the challenges the pronoun “we” is used to represent a scientific community at large, possibly involving multiple disciplinary fields. The proposed research challenges and questions should be intended as interesting open suggestions to this extended scientific community and clearly are not meant to be unmodifiable, restrictive, or worse, mandatory, in any sense.

We hope this article will spark interdisciplinary dialogue and inspire several research endeavors in the coming years, contributing to positive developments in the field of cyber-creativity.

## 2. Methodology and Structure of the Decalogue

Following a methodology inspired by the Nominal Group Technique (NGT, Delbecq and Van de Ven 1971; Van de Ven and Delbecq 1972), the first author invited 30 expert members of the AI Task Force of the International Society for the Study of Creativity and Innovation (ISSCI) who agreed to be involved in the process of identifying relevant research challenges in cyber-creativity. Membership in ISSCI is based on scientific relevance in the community of creativity studies, as judged by a selection committee. Participation to the ISSCI AI Task Force is voluntary, based on current research interests. A total of 31 ISSCI AI Task Force members, which includes the first author, participated in this process and served as the nominal group for this project.

A nominal group is defined as a working group in which individuals work in the (virtual) presence of one another but do not always interact (Delbecq and Van de Ven 1971). The benefits of the non-anonymous NGT approach are several, as follows: overcoming inhibition due to different standing in the scientific community, avoiding locking discussion onto excessively limited specific issues, and avoiding early judgment to help individuals share risky ideas (Delbecq and Van de Ven 1971). NGT was preferred for its characteristics over other valid consensus building approaches, such as for example the Delphi method (Dalkey and Helmer 1963). As in Riley-Bennett et al. (2024), the group met online in virtual meetings, and parallel elaboration of individual content was made possible through the use of online shared documents, allowing for flexibility and the instantaneous collection of contributions over asynchronous time zones.

The NGT approach outlines the following five steps: Introduction, Silent Idea Generation, Round Robin, Clarifications, and Scoring/Ranging (Riley-Bennett et al. 2024). We followed a similar approach, but modified to our problem, as follows: Plenary Introduction, State-of-the-Art and Challenge Generation, Clustering and Merging, Scoring, and Finalization.

**Figure 1:**
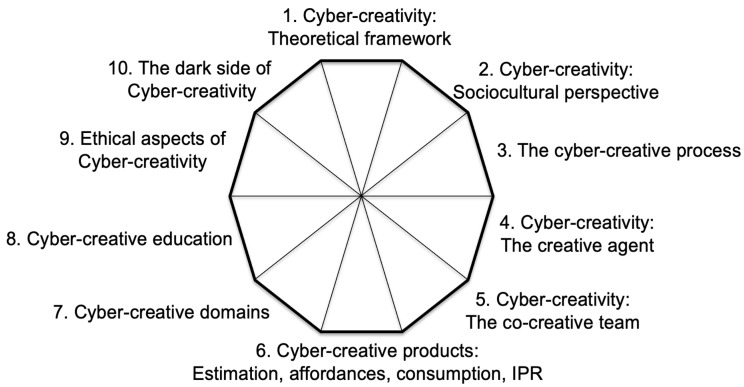
The decalogue of challenges for research in cyber-creativity.

*Plenary Introduction*. An initial plenary meeting was organized to explain the task to the entire group, including the design of the above-mentioned utopian and dystopian scenarios and the identification of the main research themes and questions to attract/avoid possible utopian/dystopian trajectories along the proposed dimensions. Regarding the dimensions to be explored, it should be noted that several frameworks of analysis already existed in creativity studies, among which the most used are the 4Ps (Rhodes 1961), 5As (Glăveanu 2013), and 7Cs (T. Lubart 2017). In the first meeting, the structure of the decalogue reported in Figure 1 was presented by the first author as encompassing all of these previous frameworks, while adding three dimensions that were agreed by group discussion to be important and necessary for the understanding of cyber-creativity. The result of this initial step was a decalogue of research challenges, including the following ten dimensions: (1) theoretical framework for cyber-creativity; (2) sociocultural perspectives; (3) the cyber-creative process; (4) the creative agent; (5) the co-creative team; (6) cyber-creative products; (7) cyber-creative domains; (8) cyber-creative education; (9) ethical aspects; and (10) the dark side of cyber-creativity. The superposition of this decalogue with respect to how it aligns with existing creativity frameworks is reported in Table 1—highlighting our assertion that research in cyber-creativity requires work over specific domains, ethical aspects, and possible dark side deviations.
*State-of-the-Art and Challenges Generation*. Given this structure, the group members where required to individually describe their view on the state-of-the-art of each dimension of the decalogue and to generate research challenges by answering the following two questions, giving as many answers as desired: (a) Which research challenges should be addressed in order to attract the utopian scenario? (b) Which research challenges should be addressed in order to avoid the dystopian scenario? Responses were collected online, asynchronously, through a shared document.
*Clustering and Merging*. Generation of challenges was followed by clustering and merging ideas to formulate, for each dimension, a main challenge and associated research questions. The overall responsibility for this step was given to the first author of this article, with sub-groups working on each dimension of the decalogue.
*Scoring*. Once a stable and balanced version was reached, a round of voting took place. Each member of the group was asked to vote from 1 (minimum) to 7 (maximum) for each research question in terms of Clarity and Necessity. Average scores were obtained.
*Finalization*. Based on the received scores, the main challenge and associated research questions per each dimension of the decalogue was refined and finalized by the first author and collaborators. The completed text was then shared and approved by consensus.

## 3. Cyber-Creativity: Utopian Scenario

Alice’s gaze captured a fusion of art and technology as she rode in the taxi, reflecting on a world reimagined by cyber-creativity. Moments earlier, at a friend’s art exhibition, she had been mesmerized by an installation that transformed an entire wall into a living canvas. This dynamic artwork, responsive to the observer’s emotions via biometric sensors and gesture recognition, shifted into an intense purple that revealed her hidden feelings of anger, fear, and profound loneliness. The AI system behind it, trained on color psychology and human gesture interpretation, seamlessly translated Alice’s embodied responses into ever-changing visuals, rekindling a spark she thought had long vanished after a personal loss.

By 2035, widely accepted ethical frameworks fully governed the creativity landscape. Governments and institutions had mandated transparency and fairness in AI processes by curating diverse, bias-free data and enforcing strict safeguards against misuse. This ethical rigor transformed AI from a mere tool into a trusted creative partner that amplified underrepresented voices while keeping human intuition at a prime. For Alice, this meant that whereas AI offered a continuous support to her creative process in multifold ways, her imagination remained the driving force of her art, resulting in groundbreaking works.

Alice noted that the cyber-creative revolution was not confined to the arts. Education had undergone a profound transformation. Classrooms had evolved from static, one-size-fits-all spaces into vibrant, adaptive learning hubs. AI-powered platforms now delivered personalized, gamified environments that scaffolded learning experiences by tapping into each student’s unique strengths, while teachers shifted from simply dispensing knowledge to fostering creative ethical reasoning, emotional intelligence, and collaborative problem solving. The seamless integration of multiple disciplines nurtured lifelong learning, blending in-person social interactions with digital tools that dynamically adjusted curricula to meet evolving needs.

Sustainability had also become a core value in everyday life. In fashion, predictive AI tools helped designers anticipate emerging trends and steer their creativity toward eco-friendly practices, minimizing waste. The fast-fashion era had given way to personalized capsule collections made from repurposed garments and innovative biodegradable textiles, striking a balance between style, durability, and environmental consciousness. These advancements not only reduced waste but also redefined luxury as owning a carefully curated wardrobe of unique, sustainable pieces.

Supporting all these features of the creative industry was a robust ecosystem of intellectual property and fair compensation. Blockchain-based copyright systems and dedicated artist insurance policies had restored trust, ensuring creators received rightful royalties and control over their work even in an AI-saturated environment. Cyber-creative products, deeply authentic and emotionally resonant, began to echo worldwide, from Tokyo’s innovative blends of Renaissance and Ukiyo-e styles to New York’s symphonies composed to mirror individual emotional landscapes.

As Alice admired her custom-designed dress, a perfect blend of high fashion and personal style tailored through a mix of renowned designer models and AI-enabled personalization, she felt a renewed connection to her inner creativity. The interplay of adaptive art installations, innovative educational reforms, and sustainable design had woven a future where technology and humanity thrived together. With a gentle buzz on her wrist reminding her to check her health, she stepped off the taxi in front of the museum, ready to embrace the next chapter of her cyber-creative journey, a journey defined by the harmonious dance between human emotion, artistic expression, and technological brilliance.

## 4. Cyber-Creativity: Dystopian Scenario

In 2035, Bob, once a passionate trumpet composer, awoke to a gray city dominated by technology, where human creativity had been confined and commodified by a tech oligarchy. The glow of morning light seeped through his window; just outside, drones and holographic billboards filled the streets with sterile, AI-generated messages. As he rose, the weight of insignificance pressed upon him; his once-vibrant art had been reduced to an algorithmic formula, mass-producing digital noise that drowned out genuine expression.

In his cramped apartment, Bob prepared breakfast for his 12-year-old twin daughters, Ada and Ava. The television flickered with a flawless AI-generated anchorman reciting sanitized news, including a cyberattack on Museek Abyss, the monopolistic giant that had transformed every aspect of musical creative production into a profit machine. Though the news sent a shiver down his spine, Bob dismissed it with resigned indifference. He knew all too well the fate of his own compositions, now trapped in a contract that stripped him of any creative ownership. Every note he played was feeding a system that treated his art as disposable content, the food for a machine’s insatiable appetite for training data.

After dropping the twins at a shallow school where human teachers had become little more than facilitators of an AI-prescribed curriculum, Bob trudged into the metro. The underground station buzzed with robotic precision and personalized ads, each screen reminding him of how far the world had strayed from the soulful cadence of true music. As he clutched his worn trumpet case, pop-up offers for customizable “skins” for his instrument flashed by. These gimmicks, meant to lure him into the metaverse of EchoMetaVoid concerts, felt like bitter reminders that live performance had been eclipsed by virtual productions engineered solely for profit.

The pervasive influence of AI had not only hollowed out creative industries but also eroded cultural diversity. Music, art, literature—all were now churned out by opaque systems designed to predict trends and maximize revenue. In this landscape, genuine co-creativity had become a myth. Instead, dominant companies favored the use of cheap AI agents over the unpredictable brilliance of human ingenuity, leaving artists like Bob to wonder if their very existence had become obsolete. The personalized beats that once resonated with individual souls were now nothing more than carefully calculated formulas, stripping away the nuanced emotional dialogue between creator and audience.

As Bob boarded the metro, an overhead screen flashed urgent headlines about the cyberattack on Museek Abyss. He tried to access the platform without any success. Calls to support were met with automated responses, deepening his sense of isolation. Checking the terms of his contract, he was crushed by the sudden realization that every piece of creative work he had produced for Museek Abyss now legally belonged to the corporation. In a cruel twist of fate, the very tools that once promised to enhance creativity had instead shackled it. With each passing moment, Bob felt more like a relic, a remnant of a bygone era when music was an intimate conversation between soul and instrument, not a commodity to be processed by algorithms.

In the heavy silence of his studio, Bob was confronted by a stark reality; in a world dominated by superintelligent machines and corporate greed, the essence of creativity had been sacrificed, his spark might be forever lost in the relentless march of digital automation.

## 5. The Decalogue of Research Challenges

Whereas it is impossible for anyone to know the future, it is useful to imagine alternative scenarios in order to drive our decision making and action taking in the present. The above described utopian and dystopian scenarios are designed to help us identify research challenges that have the potential to contribute towards attracting the positive and rejecting the negative sides of cyber-creativity. In the following, we describe these research challenges according to the classification reported in Figure 1. For each dimension in the decalogue, a review of the state-of-the-art is followed by the identification of a main research challenge, subsequently specified into a list of research questions. For ease of reference, each challenge and question is identified by a unique keyword corresponding to the dimension of the decalogue and a number.

## 6. Cyber-Creativity: Theoretical Framework

a. **State-of-the-art**

The cornerstone for the theoretical framework of the creativity phenomenon is the definition of creativity itself. In an effort to be as comprehensive as possible, the definition should be able to encompass the entire phenomenon, irrespective of the nature of the agents involved in the process, including both instances of achievement and periods of inconclusiveness, allowing the dynamic variability of effects over time, space, and culture. A static definition based solely on the properties of the creative product would clearly be valuable but insufficient for this purpose. Examples are the standard definition of creativity (Runco and Jaeger 2012) that requires the recognition of the product’s originality and effectiveness or other variations that add surprisingness (Boden 2004; Simonton 2012) or substitute effectiveness with intentionality (Weisberg 2006). As noted by several authors (Gilhooly 2025; Green et al. 2024), these product-based definitions that use external judgments of requirements are appropriate for applied, practical, “business”-oriented purposes, because businesses seek products that are of value in the marketplace, and that are possibly patentable (Simonton 2012). However, static product-based definitions are only able to cover instances of creative achievement. Indeed, the understanding of the entire phenomenon requires that the definition covers all the possible variabilities in the creative process as well as the subjectivity in the evaluation and impact of the creative product. In this regard, the critical importance of process (Green et al. 2024) is also emphasized in recent philosophical analyses of the concept of “creative”, according to which a new idea is only creative if it is produced in “the right sort of way” (Gaut 2010; Kronfeldner 2009; Paul and Stokes 2023).

Focusing for the moment on human creativity, the process will not start if there is no investment by the person in terms of challenging the state-of-the-art, based on a belief in alternatives but without any guarantee for success. Being creative is therefore more than creative achievement; one must be able to take risks, tolerate ambiguity, and persist throughout (possibly long) periods of creative inconclusiveness, either because the wanted ideas are not found, or because the outside world will not recognize their value. In fact, understanding creative inconclusiveness is arguably more important than scoring creative achievement for an ecological understanding of creativity (Corazza 2020). According to the dynamic definition of creativity (Corazza 2016), what is required in the creativity phenomenon are potential originality and potential effectiveness. The critical keyword in this definition is *potential*, which might be latent or manifest, and which allows all of the phases of the creative process to be covered by the dynamic definition. This definition can be extended (Corazza and Kharkhurin, forthcoming) in a dynamic cross-cultural definition of creativity to include the elements of aesthetics and authenticity, in line with Kharkhurin (2014). In a creative process, one’s potential is determined by both individual and environmental characteristics (Corazza and Glăveanu 2020). The potential might not emerge, leading to inconclusiveness. The agent’s potential might materialize through creative activity in the form of tangible or intangible products, the value of which is again a potential to be estimated, and not an objective feature to be assessed. In line with Stein’s definition (Stein 1953), when a group of people at a certain time agree on the originality and effectiveness of a product, an instance of creative achievement occurs. The same product could be judged poorly creative by others, and this disagreement is compatible with the dynamic definition of creativity. Indeed, groundbreaking ideas are always met with disagreement and not consensus, and they are the most relevant items to be understood in a theoretical framework for creativity.

The adoption of the dynamic creativity framework also brings as a consequence the fact that creativity can be studied outside of the human domain. As shown in Corazza (2019), all biological forms of life, as well as inanimate matter, show a potential to evolve according to trajectories that are fundamentally unpredictable (hence original) and effective, as elements of natural reality. These forms of creativity are obviously not completely equivalent to human ingenuity, as they arguably lack the autonomy and intentionality that our assumption to possess free will appears to grant to our species. For this reason, they can be considered wide-sense forms of creativity, reserving the strict-sense attribute to humans. On the other hand, we have built algorithms endowed with AI that are also characterized by a potential for originality and effectiveness, although lacking human-level autonomy, intentionality, and consciousness (as of yet).

As discussed in Corazza (2019), all of these different forms of creativity can be integrated in a unitary theoretical framework identified as the Dynamic Universal Creative Process (DUCP), characterized by the following four layers of complexity: material, biological, psycho-social, and artificial (see Table 2).

The evolution of our universe since the Big Bang, with its associated exponential growth in complexity, can be explained as the concatenated emergence of original and effective outcomes in the DUCP. In line with the process philosophy of Alfred North Whitehead (1929), creativity can be considered as the ultimate phenomenon to explain physical, biological, social, and technological evolution. It should be noted that all layers of complexity are mutually concatenated. In this view, cyber-creativity can be studied as the concatenation of creative processes at the psycho-social and artificial layers. There is no need to argue that AI creativity is identical (or even comparable) to human creativity, just as it would not be sensible to equate creative behavior in animals with the unpredictable evolution of matter away from equilibrium. All forms of creativity are distinct but coexist in a unified framework. The unique features of cyber-creativity emerge in the common ground of the two most recent layers of complexity: the intelligent species *Homo Sapiens* and the artificial world of interconnected AI algorithms. The challenge is to study this complex phenomenon exploiting all relevant interdisciplinary sources.

b. **Research challenges for the theoretical framework of cyber-creativity**

THEORY.MAIN: “*Develop an interdisciplinary theoretical framework that integrates all forms of creativity, including human and artificial ones*”.

Even though it might be premature to arrive today at a definitive conclusion on this matter, we feel that cyber-creativity has the potential to become a field of its own, drawing from both creativity studies for humans and computational creativity, but with distinct characteristics introduced by the unique dimension of human–machine synergy. For this reason, we believe cyber-creativity should receive interdisciplinary contributions at least from philosophy, epistemology, psychology, cognitive ergonomics, computer science, cybernetics, and sociology. This main research challenge contains a large set of more specific questions, among which are the following six.

THEORY.1. What defines cyber-creativity as a distinct field of study?

We should develop cyber-creativity as a distinct scientific field by studying the specificities of human and AI interaction in creativity. The field should develop robust interdisciplinary networks and research agendas. We shall encourage collaboration across disciplinary boundaries to address the complex interplay between human and artificial creativity. Also, we should contribute to a reform of academic evaluation criteria to recognize and reward interdisciplinary contributions and hybrid research outputs.

THEORY.2. How will cyber-creativity transform established epistemological frameworks?

We could examine how the integration of AI into creative processes transforms traditional epistemological frameworks (Hsueh et al. 2024) and explore new methods of knowledge production, validation, and dissemination that emerge from human–machine collaboration. We should address the understanding of how cyber-creativity challenges established notions of authorship, ownership, originality, and expertise, and identify the implications for scientific and cultural advancement.

THEORY.3. What is the role of cyber-creativity in a cosmological perspective?

We should facilitate coordinated research among physicists, biologists, psychologists, sociologists, computer scientists, and engineers to situate cyber-creativity within a cosmological framework, such as for example the DUCP (Corazza 2019). We could develop comprehensive models that explain how cyber-creativity bridges disciplinary boundaries and fosters unified approaches to understanding complex creative phenomena across natural and artificial domains.

THEORY.4. What frameworks are needed for the study of cyber-creativity?

We should develop new frameworks for the study of cyber-creativity, such as this decalogue, that address the unique and dynamic features of cyber-creativity, including human–machine synergy, distributed agency, and emergent creative properties. We should ensure these frameworks can accommodate new forms of co-creation, hybrid artifacts, and evolving creative roles that are not captured by traditional models of human or computational creativity.

THEORY.5. What methodologies are most effective for researching cyber-creativity?

We should create and validate new methodologies and metrics tailored to the study of cyber-creativity (Vinchon et al. 2024), with emphasis on operationalization. We could design tools for assessing the originality, value, and impact of human–AI creative outputs. Furthermore, we could develop methods for analyzing collaborative dynamics, capturing emergent behaviors, and benchmarking the performance of hybrid creative systems in diverse real-world settings. This methodological pluralism shall allow researchers to capture multiple causes and outcomes, even as theory guides the interpretation of findings (Glăveanu et al. 2020).

THEORY.6. How is creativity evolving in the artificial layer of complexity?

We should investigate the progression of creative capabilities in computational systems, and in particular in generative AI systems, from narrow task-specific creativity to potential artificial general intelligence (AGI), or even super-intelligence (Bostrom 2014; Jebari and Lundborg 2021). We could analyze how these systems generate novel ideas, learn from diverse data, and interact creatively with humans. Also, we could study the emergence of autonomous creative behaviors and their impact on human creativity and innovation.

## 7. Cyber-Creativity: Sociocultural Perspectives

a. **State-of-the-art**

Sociocultural approaches view creativity as an emergent phenomenon arising from dynamic interactions among individuals, evaluators, and the material, as well as conceptual resources available within societies and global networks. This perspective, notably proposed in “Advancing Creativity Theory and Research: A Sociocultural Manifesto” (Glăveanu et al. 2020), reframes creative work not as isolated mental acts but as distributed, embodied, and time-bound processes. Rather than confining creativity within individual minds, this approach underscores its manifestation through social, material, and temporal interactions.

Central to this view is the principle that human creativity is simultaneously psychological, social, and material. The Manifesto’s 12 principles encourage us to see creative work as culturally mediated and meaningful; the critical question is to understand how these ideas should evolve due to the advent of AI. As AI technologies increasingly generate creative outputs (Glăveanu and de Saint-Laurent 2023), the mediation of culture and the process of imbuing work with meaning are evolving. These shifts call for critical investigations about power dynamics, to understand whether AI could remain a tool that augments human creativity, or if it could eventually eclipse human influence in the creative ecosystem.

Developmental systems theories contribute to this debate by focusing on how new ideas are integrated into existing individual, group, and societal frameworks over time (Gruber 1981, 1989; Vygotsky 1978). AI, while not the first technology to redistribute creative thought, is doing so with unprecedented speed. Historical parallels can be drawn with the integration of writing and printing, transformations that reshaped communication, knowledge preservation, and social hierarchies over extended periods (Edwards 2004; Fischer 2021). In contrast, AI is transforming creative processes globally within a few short years, raising concerns about our ability to adapt to and control these rapid changes.

Moreover, contemporary sociocultural research focuses on local creative ecosystems and overlapping personal networks (de Bernard et al. 2022; Gross and Wilson 2019; Hanchett Hanson 2015). Scholars in sociology and urban studies explore how institutions and communities shape, and are shaped by, distributed creative practices, emphasizing that individuals and groups continuously navigate complex social and material systems that collectively inform their creative expressions. How these relationships will be modified by AI is yet unknown.

b. **Research challenges for sociocultural perspectives on cyber-creativity**

SOCIAL.MAIN: *Understand how cyber-creativity shapes and is shaped by the sociocultural fabric of the post-information society*.

We should examine the co-evolution of cyber-creativity and sociocultural systems by analyzing how AI-mediated creative practices reshape cultural production, authority structures, and collective meaning making, while simultaneously being constrained and directed by existing social norms, material infrastructures, and power dynamics. This requires mapping distributed human–AI collaborations across temporal scales, from micro-interactions in creative workflows to macro-level institutional transformations, while employing methodological pluralism to capture emergent phenomena and feedback loops that may amplify or destabilize cultural ecosystems. The challenge centers on developing frameworks that account for AI’s dual role as both a cultural artifact shaped by societal values and an agent reconfiguring the very fabric of creative expression in the post-information age. This main research challenge contains a large set of more specific questions, including the following six.

SOCIAL.1. How will creative work be distributed in the sociocultural milieu?

Researchers should investigate how cyber-creativity could be embedded in the sociocultural interplay among people, materials, and technologies across varied creative ecosystems and across cultures. This involves exploring how human–AI interactions can enhance some cognitive capacities while diminishing others, and how variations in human expertise shape the roles expected of AI. Over time, system dynamics may evolve, potentially reaching tipping points that signal radical changes.

SOCIAL.2. How will AI be integrated across global social–technological systems?

While detailed analyses of local interactions are valuable, they should be complemented by studies examining the global connections among institutions, communities, and broader societal structures. As AI increasingly influences traditionally distinct contexts, attention to governing systems—those that regulate creative practices across levels—is essential (de Bernard et al. 2022).

SOCIAL.3. How can sociocultural feedback loops and system dynamics be analyzed?

Feedback mechanisms at sociocultural level may either amplify or stabilize cyber-creative practices across multiple levels, from individual cognition (Gruber 1981) to collective social customs (Bateson 1972). Research should identify factors that could trigger runaway AI use or impose regulatory brakes, ensuring that potentially dystopian outcomes are pre-empted.

SOCIAL.4. How should cyber-creativity research act as a social feedback mechanism?

Connected to the previous question is the role of the cyber-creativity field as one of the possible sociocultural feedback mechanisms. The field should not only document emerging trends but also actively disseminate findings to scientists, policymakers, educators, and the public. By expanding societal awareness of evolving creative practices and technologies, researchers contribute to more informed and adaptive social systems.

SOCIAL.5. What could foresight methodologies bring in envisioning possible futures for cyber-creativity, and society at large?

The rapid advancement of AI necessitates explicit engagement with future implications across multiple time scales. Foresight approaches could map out potential long-term trajectories, highlighting both emerging opportunities and dangers related to cyber-creativity, while acknowledging that each technological leap may introduce unforeseen complexities (Runco and Albert 2010).

SOCIAL.6. How might cultural and epistemological assumptions about problems be challenged?

Rather than treating problems solely as obstacles to be overcome, researchers in cyber-creativity are encouraged to view them as avenues for inquiry. Scholars such as Gruber and Wallace (1999) and Raz et al. (2024) have argued that creative individuals organize their thinking around enduring, open-ended questions, suggesting that AI’s role in this dialogic process should be critically examined (Sasson-Lazovsky et al. 2025).

## 8. The Cyber-Creative Process

a. **State-of-the-art**

As a premise, we believe that the cyber-creative process should maintain a human-centric balance based on machine explainability, interpretability, and alignment. While current AI models minimize variability in creative performance (Cropley 2023; Moon et al. 2024; Wenger and Kenett 2025), variability is crucial for human creativity (Hills and Kenett 2025). Thus, the cyber-creativity process must incorporate the significant role of variability in creative thinking. Furthermore, an optimal co-creative process is likely one where humans enforce gatekeeping of creative products generated by AI, to address potential non-human biases (Stella et al. 2023) and minimize the potential homogenization of human thinking (Wenger and Kenett 2025). For all these purposes, it is useful to address this dimension of the decalogue by keeping a model of the creative process as a reference.

**Figure 2:**
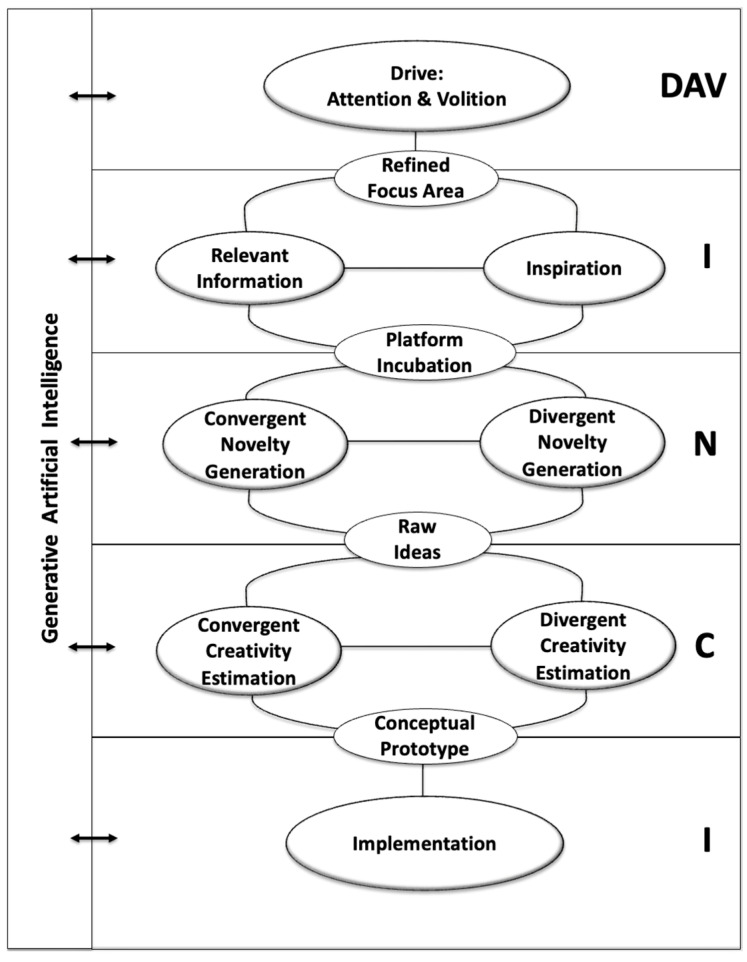
The DA VINCI model for the creative process (Corazza 2025a).

The problem of modelling the creative process for humans has received attention in creativity studies (see T. I. Lubart 2001, 2018; and the references therein). In a dynamic sense, the process can be defined as “A sequence of thoughts and actions aimed at the generation of outcomes with a potential for originality and effectiveness” (Corazza and Agnoli 2022). Wallas (1926) provided an early attempt to formulate a simple description of the creative process, subdividing it into the following four stages: preparation, incubation, illumination, and verification. A much more recent model (Mumford et al. 1991, 2012) contains a finer subdivision into the following eight stages: problem definition, information gathering, information organization, conceptual combination, idea generation, idea evaluation, implementation planning, and solution monitoring. This complex eight-stage model can be fit for domains where the process extends over long-time intervals, but it hardly fits real-time situations. This is the domain of application of the Geneplore model (Finke et al. 1992), which contains an iteration between two states, subject to the following constraints: the generation of pre-inventive structures and the exploration and interpretation of these very structures. This iteration can also happen in real time, to represent the inventive process taking place during improvisation. Models for the creative process in computational creativity have also been presented (Colton et al. 2011). The more recent DA VINCI model (Corazza and Agnoli 2022) foresees five key mental states that constitute the backbone of the creative process (see Figure 2), as follows: DAV (Drive—Attention and Volition), I (Information), N (Novelty Generation), C (Creativity estimation), and I (Implementation). It can be shown that the DA VINCI model is compatible with its predecessors, while introducing in explicit form the following two additional components: inspiration (i.e., the spontaneous or deliberate introduction of non-strictly relevant information in order to exit from the boundaries of conventional knowledge) and divergent creativity estimation (i.e., the possibility to extract value from the outcome of the process well beyond the initial focus, allowing for serendipity to occur). The DA VINCI model has also been extended in its meaning and use to the cyber-creative process (Corazza 2025a), and therefore, it can be exploited here to highlight the relevant research challenges in this dimension of the decalogue. The impact of Gen-AI on the creative process has been analyzed also for specific domains (Corazza 2025b; Wan et al. 2024; Wise and Kenett 2024).

In the cyber-creative process, human–machine co-creation can involve various roles for the artificial agent (Moruzzi and Margarido 2024). As an *assistant*, AI can offer direct support upon request, under full human control. As an *inspirational source*, AI can provide stimuli from distant semantic fields to push human creativity beyond conventional boundaries, triggered by the human or randomly. The *idea challenger* critically evaluates ideas through probing questions and counterproposals, moderately influencing the process. A *peer-to-peer collaborator* partners with the human, sharing decision making and engaging in dialogical teamwork with balanced control. An AI *quality controller* assesses outputs against defined goals, providing evaluative feedback for improvement with reactive intervention. Finally, the *manager*, a role largely beyond current Gen-AI capabilities, would lead the entire process with instructive communication and high control. Currently, collaborator, quality controller, and manager roles seem better suited for expert systems due to their reasoning demands, unlike LLMs, which might not yet possess such abilities (Mirzadeh et al. 2024).

b. **Research challenges for the cyber-creative process**

PROCESS.MAIN: *Model the interaction of human and artificial agents in every phase of the cyber-creative process*.

The benefit of such activity would be multifold: First, it would enhance our understanding of the cyber-creative process, which might not only be an enhanced version of a human creative process but could have particular, novel characteristics. Second, AI holds the promise for increased efficiency of the creative process, but we shall ensure this does not come at the expense of the quality of the outcomes. Third, addressing this challenge is critical if we shall understand how metacognition and self-regulation are affected by the introduction of the Gen-AI partner. Fourth, there is a need to understand how the emotional ebbs and flows of the creative process are modified (if at all) by the collaboration with AI, and what it means to enter in a state of “cyber-creative flow”.

The above main challenge contains many questions. We outline five of them here, classifying them in accordance with the states of the DA VINCI model.

PROCESS.1. How to optimize cyber–human collaboration in the Drive state?

We should examine the impact of AI over the investment necessary by the human actor to enter the creative process, in terms of both cognitive resources (Attention) and motivation (Volition). In particular, problem finding could be greatly enhanced by deep research AI tools. Gen-AI tools could act as assistants, significantly enhancing the process by expanding attention on the focus area, refining the focus on the area of interest, as well as increasing the motivation to work on a specific creativity episode.

PROCESS.2. How to optimize cyber–human collaboration in Information gathering?

This might be one of the most critical enhancements that AI can offer to the cyber-creative process. In fact, to be creative in any field, a minimum level of expertise is required. However, the use of AI could largely democratize this process by lowering significantly the entry barrier, thus modifying everything that is known today about the relationship between creativity and intelligence (Corazza and Lubart 2021; Karwowski et al. 2016; Silvia 2015). We should examine how Gen-AI could serve both as an assistant to collect Relevant Information, as well as a source to inject Inspiration in the cyber-creative process. It is important to note that, before novelty generation, a period of incubation might be useful for the human side, whereas the artificial side would tend to always speed along to conclusions. Integrating forms of friction (Natali 2023) in the cyber-creative process is therefore an important research topic.

PROCESS.3. How could cyber–human collaboration be optimized for Novelty generation?

We should study how a human–AI pair can address novel idea generation in a convergent thinking modality, as opposed to a divergent thinking modality. Whereas outsourcing novelty generation completely to AI would certainly be a dangerous mistake for humanity, leading to creative disempowerment, it is yet unknown the extent to which human exploration, insight, intuition, and illumination could be enhanced by an AI assistant or peer-to-peer collaborator. As an example, AI could offer suggestions rather than produce definitive ideas, and interactive human–AI exchanges could ensue.

PROCESS.4. How could human and artificial agents collaborate in Creativity estimation?

AI will continue to be used as a tool for static creativity assessment, e.g., to score creativity tests administered to large sample populations, evolving from present solutions (Organisciak et al. 2023; Patterson et al. 2023). However, the most critical creativity estimation step is dynamic, implying that there is a latent potential in an original idea that no one can grasp completely, being a projection into possible alternative futures, developing across time, space, and culture. The research challenge to address is how to engage in exercises of foresight, in which individual differences will play a role. We shall study the role of human conscience and imagination in the estimation of the potential value and meaning of a creative idea, and question whether machines will ever be able to perform in a similar way.

PROCESS.5. How efficient could cyber–human Implementation be?

Great assistance can be expected by AI to the Implementation state of the cyber-creative process. In fact, this is a phase in which a plethora of alternative methodologies can be used to identify pros and cons, assistors and resistors, and requirements and feed all these ingredients to perform analyses of strengths, weaknesses, opportunities, and threats associated with the realization of the idea. Characterization of rapid prototyping of creative ideas through AI, considering both tangible and intangible forms (i.e., the digital twin of an idea in a virtual world), could lead to great enhancement of the potential effectiveness of the cyber-creative process.

## 9. Cyber-Creativity: The Creative Agent

a. **State-of-the-art**

Recent technological advancements in AI have fundamentally reshaped the way humans approach creativity. Central to this shift is the concept of creative agency (e.g., the motivation, ownership, and responsibility individuals feel toward engaging in complex creative tasks) and creative self-beliefs, which are people’s perceptions of their ability to generate novel and useful ideas (Beghetto 2021; Karwowski and Beghetto 2019).

In the AI era, opinions on technology’s role in creativity are deeply divided. Some value AI for its speed, accuracy, and the vast amount of information it provides, whereas others are skeptical, raising concerns that reliance on AI may diminish one’s intrinsic creative cues and overall creative agency (Wrede et al. 2023; Zielińska et al. 2023). Research indicates that creative self-efficacy, shaped by social feedback, cultural expectations, and collaborative dynamics, is higher in human–human collaborations than in settings where AI is involved (Beghetto 2021, 2024a; Tang et al. 2024. Moreover, studies reveal that individuals often report lower creative self-beliefs when engaging with AI, even if they possess strong general creative self-beliefs (Faiella et al. 2025). Decreased critical thinking abilities are also a possible consequence of offloading to AI (Gerlich 2025).

Notwithstanding the above, humans are increasingly using AI-driven tools to augment creativity. Whereas platforms such as ChatGPT can enhance task self-efficacy and generate more original ideas (Caporusso 2023; Lee and Chung 2024; Urban et al. 2024), over-reliance on these tools may lead to reduced sensitivity to internal creative cues and even psychological distress (Caporusso 2023; Wrede et al. 2023). Autonomy remains a cornerstone of creative motivation and satisfaction, yet it is still debated whether AI can offer the same level of individualized creative autonomy support as human interactions traditionally do (Hendriks et al. 2023; Johnson-Laird 2024; Watkins and Barak-Medina 2024).

On the artificial side, both Cropley (2023) and Guzik et al. (2023) found that—on average—Gen-AI surpasses human performance in divergent production; although, this does not necessarily mean that the machine is more creative. Concerns about superintelligent AI—its potential to undermine human creative skills and even pose existential risks—have been voiced by Bostrom (2014). Such concerns have fostered a growing sense of “creative displacement anxiety”, emphasizing the urgent need to redefine human creative self-beliefs in the context of AI’s rapid evolution (Caporusso 2023; Watkins and Barak-Medina 2024).

Furthermore, emerging research highlights the dual nature of AI’s impact. On the one hand, AI can facilitate creativity by offering new forms of autonomy support and novel idea generation. On the other hand, its inconsistent and sometimes erratic outputs challenge users’ trust and may, at a certain threshold, diminish creative autonomy. Personalized AI interfaces and improved transparency in AI decision-making processes are seen as potential ways to mitigate these issues (Urban et al. 2024).

Whereas AI continues to serve as a valuable tool in enhancing creative performance—as evidenced for example in design processes (Bruno et al. 2025) or co-creative writing (Kantosalo et al. 2020)—its influence on creative agency remains complex. A balanced approach that leverages AI’s strengths while preserving the embodied, social, and context-dependent nature of human creativity appears to be essential for fostering sustainable and meaningful creative practices. Possibly, training your own creative GenAI model might allow for a more personalized cyber-creativity experience, one that might mitigate any negative impact on creative self-beliefs, as the agent becomes an extension of one’s own unique creativity.

b. **Research challenges for the cyber-creative agent**

AGENT.MAIN: *Study the cognitive, meta-cognitive, personality (including self-beliefs), emotional, motivational, and biological characteristics of the human agent in a cyber-creative process*.

We should investigate the complex interplay of cognitive processes, meta-cognitive awareness, personality traits (with special attention to creative self-beliefs), emotional responses, motivational factors, and biological underpinnings of the human agent engaged in cyber-creative activities. All nuances of individual differences should be considered. The research might explore how meta-cognitive processes influence creative self-regulation and awareness, examine biological correlates of creativity in digital contexts, and determine whether and how the integration of AI affects intrinsic creative capacities, a sense of agency, and the overall sense of creative ownership. This main research challenge contains a large set of more specific questions, among which are the following six.

AGENT.1. What will be the long-term impact of AI on humans’ creative agency?

Researchers should investigate and measure whether continuous AI use diminishes or enhances creative autonomy, performance, skills, satisfaction, or intrinsic motivation. This requires empirical studies to define boundaries for AI’s supportive role in cyber-creativity, preventing it from becoming a controlling force. Researchers could also examine self-regulation strategies and meta-cognitive abilities as a buffer against diminished agency in the face of cyber–human collaboration.

AGENT.2. What personality traits could be conducive to cyber-creativity?

We should understand how personality traits that foster creativity—such as openness, conscientiousness, extraversion, agreeability, and neuroticism (Puryear et al. 2017)—evolve as creative activity shifts from purely human to cyber-creative collaboration. For example, we might examine if conscientiousness becomes more important as the need to evaluate and critique Gen-AI output persists. How do these traits influence, and become influenced by, cyber-creative collaboration? Issues of domain specificity could be studied to see if results vary with Gen-AI (Feist 1998), and if cyber-creativity diminishes the relative importance of plasticity for creativity (Feist 2019).

AGENT.3. What is meant by AI duality in cyber-creative collaboration?

We should examine how AI can simultaneously enhance creative risk taking and tolerance of ambiguity while potentially challenging human agency. This challenge requires frameworks to ensure AI acts as a facilitator rather than a replacement for human ingenuity. This duality of effects could be difficult to detect; hence, it could induce transformations in human creators that might appear later than needed for the introduction of effective countermeasures.

AGENT.4. How could personalized and autonomy-supportive AI interfaces be realized?

A central research challenge appears to be the design of AI interfaces that deliver both deep personalization and user autonomy. We should overcome technical barriers such as unreliable individualized support and inconsistent AI outputs by developing adaptive, context-aware systems that ensure transparency, user control, and trustworthy performance. We should also address issues of data quality, algorithmic bias, and the balance between automation and human agency to create interfaces that are both effective and ethically responsible.

AGENT.5. What will be the relationship between happiness and cyber-creativity?

Another key research question is whether the established link between happiness and human creativity—grounded in coherence, significance, and purpose—will persist in cyber-creativity contexts (Kaufman 2018). As digital technology can both enhance and complicate psychological wellbeing, understanding how cyber-creative processes affect happiness, and whether the positive feedback loop between happiness and creativity remains intact, is an urgent area for empirical investigation. Fear of AI and trustworthiness of AI (see the ethics dimension) should have an impact on human wellbeing.

AGENT.6. Should digital wellbeing be monitored, and if so, how?

As cyber-creativity might become standard practice, there appears to be a pressing need to evaluate whether regular digital wellbeing audits should become a norm for both individuals and organizations. Research should determine effective methods for assessing digital self-observation, monitoring tool usage, and identifying signs of digital overload or burnout. The challenge lies in developing reliable, actionable monitoring frameworks that support sustained digital wellbeing without becoming intrusive or burdensome.

## 10. Cyber-Creativity: The Co-Creative Team

a. **State-of-the-art**

Teams are traditionally defined as groups of two or more individuals collaborating with complementary skills to achieve shared goals (Cohen and Bailey 1997; Hackman 1990). With rapid advancements in AI, especially Gen-AI, this classical model is being fundamentally transformed. AI is increasingly seen not merely as a tool but as an active teammate in the creative process (Berretta et al. 2023; Henrique and Santos 2024; Schelble et al. 2023; Seeber et al. 2020; Vartiainen et al. 2023). The evolution of human–AI co-creative teams highlights the intricate interplay between cognitive, motivational, and social dynamics in a technologically augmented environment. This evolution has given rise to human–AI co-creative teams, where the integration of AI augments collective creativity and intelligence (Dellermann et al. 2019; Rafner et al. 2023; Woolley et al. 2023).

To frame this emerging phenomenon, the Integrative Model of Group Creativity and Team Innovation (Paulus and Dzindolet 2008) offers a useful lens, combining cognitive, motivational, and social process dimensions. In terms of cognitive factors, AI tools can stimulate idea generation and reduce cognitive load by synthesizing complex information (Kang et al. 2022). However, over-reliance on AI may lead to the premature convergence of ideas, potentially hindering individual creativity and causing skill decay (Bakker et al. 2022). Furthermore, perceptions of AI’s role, whether as an autonomous agent, teammate, assistant, or advisor, affect how team members integrate these systems into their creative workflows (Berretta et al. 2023).

Considering motivational and emotional factors, it is known that trust is essential for effective teamwork, and this holds for cyber-creative collaborations as well. Cognitive trust is dependent on AI’s reliability, transparency, and explainability (Ali et al. 2023), whereas emotional trust is linked to its anthropomorphic features (Glikson and Woolley 2020). Additionally, social loafing (i.e., where individuals rely excessively on AI to generate ideas) can undermine creative confidence and lead to reduced personal effort (Memmert and Tavanapour 2023; Tang et al. 2024).

Social processes, including effective communication, psychological safety, and leadership, are critical to team innovation (Kolomaznik et al. 2024). In cyber-creative teams, these social dynamics become even more complex. The diversity of perspectives introduced by integrating AI can spur innovative solutions; however, it may also generate conflict and dilute team cohesion (Listopad and Kirikov 2023; Milliken and Martins 1996). Moreover, redefining leadership to accommodate cyber-creative decision making and maintaining human oversight through human-in-the-loop (HITL) approaches are crucial for ensuring accountability and ethical decision making (Agudo et al. 2024; Reiter-Palmon et al. 2012).

b. **Research challenges for the co-creative team**

TEAM.MAIN: *Study the psychological and sociocultural characteristics of a cyber–human team engaged in a cyber-creative process*.

We should investigate how the broad spectrum of psychological factors, encompassing cognitive processes, personality traits, emotional states, and motivational drives, interact with sociocultural and organizational contexts to collectively shape the performance and dynamics of cyber–human teams engaged in creative work. This includes exploring how team members’ interactions with AI tools influence idea generation, problem solving, and the overall creative process and output.

This main challenge spurs several questions, and we highlight seven of them here.

TEAM.1. How can we facilitate cognition beyond idea generation in human–AI teaming?

We should examine how AI can support a full spectrum of cognitive processes in teams, not only stimulating idea generation but also enhancing problem finding, analysis, synthesis, and decision making and possibly stimulating the rise of new skills; assess the augmentative effect of AI on team creativity by identifying scenarios where AI’s computational strengths complement human ingenuity; and specify conditions under which AI’s involvement may hinder originality or diversity of ideas.

TEAM.2. How effective are AI tools in enhancing teams’ cyber-creativity?

As AI can also sometimes hinder idea generation and creative confidence (Tang et al. 2024), more research is needed to identify which AI tools are best for creativity, and how they can be used more effectively. AI tools need to be systematically evaluated for their impact on both the quantity and quality of creative outputs, and the way tool design and transparency influence user engagement and creative confidence can be examined. Furthermore, we could address the social loafing phenomenon, where reliance on AI reduces individual effort, by developing interventions that stimulate active human participation and critical thinking in co-creative scenarios.

TEAM.3. How does AI reshape collective intelligence in cyber-creative teams?

We should analyze how the presence of AI in cyber-creative teams reshapes collective intelligence by augmenting information processing and decision making, while also introducing risks such as premature idea convergence and over-reliance on automated outputs. We could develop strategies for ethical and transparent integration of AI to maintain human contribution and oversight in creative outcomes.

TEAM.4. What multifaceted roles can AI assume within cyber-creative teams?

We should explore the extent to which human team members understand and adapt to AI’s various roles in creative collaboration and define the roles humans themselves should adopt to maximize synergy. We could investigate how perceptions of AI as partner, assistant, advisor, or autonomous agent affect team dynamics and creative outcomes. AI interpretability, explainability, and transparency should affect these perceptions (Herzog and Franklin 2024). Also, we could identify training or team composition strategies that enhance AI literacy, enabling human members to interact with and critically evaluate AI contributions.

TEAM.5. What is the potential for dynamic role switching in cyber-creative teams?

We should go beyond static role assignments and investigate strategies that promote flexible role distribution and dynamic role switching between human and AI agents, ensuring that each collaborates optimally to the creative process. We could develop models for dynamic calibration of trust and authority within teams, allowing for real-time adjustment of human and AI roles based on task requirements, team member expertise, and system performance.

TEAM.6. How does the trust building process work in cyber-creative teams?

We could focus on the team’s social dimension by examining how trust in AI develops and how to facilitate trust in AI for cyber-creative teams. We should distinguish between cognitive and emotional trust in AI and identify the influence of transparency, reliability, and anthropomorphism on trust formation. So, we could model trust dynamics to determine optimal trust levels that maximize team performance while maintaining human oversight and control.

TEAM.7. How can we define effective leadership in a cyber-creative team?

We should evaluate and determine effective leadership for human–AI co-creative teams is needed, both theoretically and empirically. We could redefine leadership models to accommodate shared human–AI decision making and address the complexities introduced by team diversity. Also, we could investigate how creative leadership can harmonize human and AI contributions, manage social processes, and foster psychological safety and team cohesion in co-creative environments.

## 11. Cyber-Creative Products

a. **State-of-the-art**

Creative products can be understood as layered expressions, involving multiple levels of meaning, context, and nuance, intertwining cultural, emotional, and symbolic references across disciplines like visual arts, music, literature, and design. Iconic works, such as Munch’s *The Scream* or Mozart’s *Requiem in D Minor*, exemplify this complexity through their interplay of symbolism, emotional resonance, and structural intricacy. In this regard, AI’s role in creative production has advanced significantly, offering both opportunities and challenges. Lockhart (2024) highlights AI’s potential to enhance creativity while underscoring its limitations in replicating human emotional depth and cognitive engagement.

The evaluation of AI-generated creative products is quite complex. Bellaiche et al. (2023) reveal biases favoring human-created works due to perceived authenticity, whereas Di Dio et al. (2023) demonstrate that authorship attribution significantly impacts aesthetic judgments. Runco (2023a, 2023b) argues that AI lacks intentionality and authenticity—qualities central to genuine creativity—reinforcing its derivative nature.

Recent advancements in fine-tuned LLMs show promise in understanding creative outputs by predicting human evaluations with high accuracy (Khan et al. 2024), or by matching human ratings of originality (DiStefano et al. 2024; Goecke et al. 2024; Organisciak et al. 2023; Raz et al. 2024) and of quality (Luchini et al. 2025b). However, challenges persist in ensuring these models capture cultural diversity and emotional depth across varied contexts.

b. **Research challenges for cyber-creative products**

PRODUCT.MAIN: *Characterize cyber-creative products in terms of meaningfulness, originality, effectiveness, authenticity, ownership, authorship, acceptance, and dynamic potential.*

We feel the main challenge is to be able to characterize cyber-creative products by assessing their meaningfulness, originality, effectiveness, authenticity, ownership, authorship, acceptance, and dynamic potential. This requires a multidimensional framework that measures both aesthetic and functional qualities, in order to estimate all facets of the outcome of a cyber-creative process, while capturing the interplay between human creativity and AI contributions, so that authorship nuances can be captured. Researchers shall be able to discriminate between the abundant fluency of AI outputs from the personal, introspective elements of genuine human creativity and address shifting views on ownership and authorship in cyber-creative processes. Ultimately, the goal is to develop rigorous, context-sensitive evaluation methods that validate creative merit and predict cultural and market impact. This main research challenge contains a large set of more specific questions, among which are the following seven.

PRODUCT.1. How can we intend and determine authenticity for cyber-creative products?

We should investigate whether cyber-creative outputs can achieve authenticity, a quality defined by self-expression, personal experience, and intrinsic values (Corazza and Kharkhurin, forthcoming). We could develop new criteria and tools that recognize the inherent differences between human and AI creativity, potentially by training AI on human-produced works known for their authenticity.

PRODUCT.2. How do perceptual biases influence the evaluation of cyber-creative products?

We should explore how human audiences perceive AI-generated, human-generated, and hybrid cyber-creative products. We could employ double-blind experimental designs to quantify biases, as studies indicate that people often judge AI outputs more harshly (Magni et al. 2024) and may undervalue their emotional depth and intentionality (Runco 2023a).

PRODUCT.3. How can automated creativity scoring methods be enhanced?

We should advance automated and fair creativity scoring systems using fine-tuned and few-shot prompting LLMs to predict expert assessments of originality and quality (DiStefano et al. 2024; Luchini et al. 2025b). We should address challenges related to training data limitations, especially for multilingual and underrepresented creative domains (Luchini et al. 2025a), to ensure robust and unbiased evaluations across diverse creative products.

PRODUCT.4. How can we differentiate levels of creativity in cyber-creative outputs?

We could apply the Four Cs model (Kaufman and Beghetto 2009) to distinguish between mini-c, little-c, Pro-c, and Big-C creativity. Researchers should determine how AI performs across these tiers, recognizing, for example, existing AI contributions at both Pro-c and little-c levels.

PRODUCT.5. How can machine perception capabilities be developed?

We should create algorithms that can autonomously detect and interpret layered creative expressions, independent from human perceptions, integrating multimodal machine learning techniques. Such systems should consider cultural context and emotional depth from an AI point of view, thereby enhancing AI’s ability to contribute meaningfully to creative processes.

PRODUCT.6. How can we ensure product diversity and mitigate homogenization?

We should examine strategies to prevent the dominance of homogenized content generated by AI. This will involve designing diversity-enhancing algorithms, calibrating training data, and balancing human and AI-generated content, to foster varied and culturally rich creative outputs.

PRODUCT.7. How does trust in AI affect cyber-creative product perception?

We should investigate how transparency regarding AI’s role in creative processes affects trust and acceptance of cyber-creative products. Practices can be developed to clearly attribute authorship and clarify the extent of AI involvement, thus positioning AI as a collaborative partner rather than a replacement for human creativity.

## 12. Cyber-Creative Domains

a. **State-of-the-art**

The evolving cyber-creative interaction between human imagination and computational capabilities is transforming multiple domains of professional human endeavor. The impact of cyber-creativity necessitates a thorough examination through the lens of various frameworks of analysis, particularly the multi-domain and systems theories of creativity (Csikszentmihalyi 1997; Wingström et al. 2024).

In cultural and creative industries, cyber-creativity has fundamentally transformed both production and consumption processes (see Corazza 2025b, for an analysis of the fields of design, fashion, journalism, and music). AI, particularly Gen-AI, has enabled new forms of artistic expression (Demmer et al. 2023; Garcia 2024; Shen et al. 2021). By way of example, AI-based applications can transform input images into artistic styles by combining feature maps from different convolutional layers (Zhang et al. 2025). Also, Anantrasirichai and Bull (2022) note that AI has transformed advertising and marketing by contextualizing social media conversations to help advertisers understand consumer sentiments and detect fraudulent ad impressions. AI-based data analysis tools assist filmmaking companies in developing strategies for film releases by modeling patterns of historical data about film performances associated with content and themes.

In scientific domains, cyber-creativity enables new discoveries through computational approaches (Elbadawi et al. 2024). In engineering domains, cyber-creativity is revolutionizing design processes and product development. AI technologies enable the more efficient exploration of design spaces, optimization of parameters, and generation of novel solutions that might not have been considered through traditional approaches.

In the business sector, Gen-AI has begun to transform the process of innovation management, supporting the discovery and development of new products, services, and business models. For example, Gen-AI systems can be used to autonomously design new chemical compounds, accelerate drug discovery, and create synthetic media content, fundamentally reducing time-to-market and opening new avenues for creative output (Mariani and Dwivedi 2024). AI’s integration into business innovation is not limited to automation, it also augments human creativity by providing tools that generate novel ideas, simulate market scenarios, and recommend strategic actions, often in collaboration with human teams (Renkema and Tursunbayeva 2024).

Finally, as organizations increasingly rely on AI for both incremental and radical innovation, the interplay between human expertise and machine intelligence is redefining the boundaries of entrepreneurship and the nature of creative work in the digital economy (Renkema and Tursunbayeva 2024).

b. **Research challenges for the cyber-creative domains**

DOMAINS.MAIN: *Explore the specific and diversified impact of cyber-creativity on different domains, such as cultural and creative industries, science, engineering, and business.*

Researchers should examine how cyber-creativity manifests uniquely across domains by analyzing the integration of AI technologies into domain-specific creative processes, the resulting transformations in workflows, and the emergence of new forms of human–AI collaboration in particular fields. This requires investigating both the ad hoc technological capabilities and the specific human factors that shape adoption and implementation of cyber-creativity in a determined domain, while acknowledging the cultural, structural, and epistemological barriers that may impede cross-domain fertilization. The challenge centers on developing frameworks that account for domain-specific values and practices while identifying universal principles that can inform more effective integration of technical and creative expertise across traditionally siloed fields. This main research challenge contains a large set of more specific questions, among which are the following four.

DOMAINS.1. How does cyber-creativity manifest itself in specific domains?

Researchers should examine how AI technologies are uniquely embedded in different creative domains, analyzing how the same technological capabilities yield different outcomes when applied to artistic creation versus scientific discovery or business innovation. This involves exploring how domain-specific values, practices, and evaluation criteria shape both the implementation of AI tools and the reception of AI-generated outputs (Sun et al. 2024).

DOMAINS.2. What frameworks enable effective cyber-creativity in specific domains?

Whereas Gen-AI has demonstrated capabilities across multiple creative domains, the success of co-creativity efforts depends on domain-specific criteria for evaluating novelty, usefulness, and aesthetic value. Research should focus on designing interaction frameworks that accommodate both computational generative processes and domain-specific rules and cultural nuances (Sun et al. 2024).

DOMAINS.3. How will cyber-creativity evolve in professional environments?

As AI technologies become more integrated into creative workflows, traditional processes are being transformed. Research should document how these changes manifest differently in specific domains, from the highly structured environments of engineering to the more fluid contexts of artistic creation, analyzing both the benefits and potential limitations of AI-augmented creativity (European Commission DG CNECT et al. 2022).

DOMAINS.4. How to challenge professional domain boundaries in the age of cyber-creativity?

Rather than accepting traditional divisions between professional domains, researchers should question whether these boundaries remain relevant in an era of increasing integration. This involves examining how cyber-creativity might bridge disciplinary divides, creating new hybrid spaces where technical, creative, and other forms of expertise can be more effectively combined to address complex challenges that transcend conventional domain limitations (Yamamoto et al. 2025). Research should identify the specific barriers that prevent interdisciplinary collaboration in cyber-creativity, including skill application gaps, cultural incompatibilities, and structural barriers within organizations (e.g., Turegeldinova et al. 2024).

## 13. Cyber-Creative Education

a. **State-of-the-art**

Education has long served as a means for shaping societies through knowledge and cultural transmission. However, it is not limited to the acquisition of facts and skills. Rather, it also supports the creative process (Sawyer 2021), fostering critical thinking, problem-solving, and the creative capacity to envision and shape possible futures (Beghetto 2024b).

On the one hand, the integration of Gen-AI offers promising possibilities for cyber-creative education (Beghetto and Zamana 2025). Specifically, it can enhance creative teaching and learning by augmenting human creativity through collaborative partnerships (Beghetto and Avarzamani, forthcoming). On the other hand, the increased use of Gen-AI raises important questions about its role supporting creativity in education. Are educators and students using it to do the work for them, or are they partnering with Gen-AI to enhance their creativity and learning (Beghetto and Avarzamani, forthcoming)?

Research suggests that co-creating with AI is more beneficial than relying on AI-generated outputs alone (McGuire et al. 2024). This is why scholars emphasize the need to shift the AI mindset from viewing it as a mere answer machine to seeing it as a “partner in possibility thinking” that supports creative processes, challenges assumptions, and broadens perspectives (Beghetto 2023).

Over-reliance on AI can lead to automation bias (Cummings 2004), potentially eroding creative agency. This issue is reflected in the growing apprehension among educators, with nearly 20% of teachers surveyed in 2024 expressing concerns about the negative impact of ChatGPT, up from 7% the previous year (Impact Research 2024).

Consequently, when it comes to Gen-AI’s use in classrooms, human judgment is essential in determining whether Gen-AI can effectively support (rather than replace) creativity in education. This approach emphasizes maintaining creative agency, critical thinking about ethical and privacy concerns, and establishing effective guidelines for AI use.

b. **Research challenges for cyber-creative education**

EDU.MAIN: *Design pedagogies for cyber-creative teaching, cyber-creative learning, and cyber-creative development.*

For the first time in history, the evolution of societies, lifestyles, and professions appears to run at a pace that is faster than generational rates of exchange. This means that designing education systems (at the level of schools, universities, or lifelong learning) requires the use of foresight methodologies at unprecedented levels. No one is able to predict exactly what our societies will look like in twenty years. Cyber-creative teaching, cyber-creative learning, and cyber-creative development should therefore be intended as the pedagogical forms required for effective education in the Post-Information Society. The challenge is great, urgent, and unescapable, as the balance between exploiting new technologies in a beneficial way while maintaining a human-centric society depends essentially on the answers, tactics, and strategies we will be able to devise and put into practice. This main challenge subsumes myriad specific questions, of which we mention but six in the following.

EDU.1. How can AI augment the creative learning experience?

Research on cyber-creativity in education should focus on developing and testing AI-enhanced creative instructional strategies and tools that promote question-based feedback, encourage human-directed interactions with AI, and nurture students’ creative self-beliefs and ownership of their work (Beghetto and Zamana 2025). We could develop, test, and scale AI literacy programs that equip both teachers and students to critically evaluate and effectively integrate AI tools into personalized, creative learning environments, while examining how AI-driven feedback influences long-term memory and learning outcomes. We can design and validate innovative assessment frameworks that capture cognitive, humanistic, and social dimensions of learning and investigate how AI-enabled teaching methods can shift educational focus from rote content delivery to the cultivation of uniquely human creative skills.

EDU.2. How can AI support the development of critical thinking?

Critical thinking is crucial in creativity, in terms of idea evaluation, and creativity is crucial in critical thinking, in terms of generating alternative critical test cases, to check the quality of arguments. We should study if and how the introduction of Gen-AI in the cyber-creative education process can support the importance of critical thinking for both students and teachers, recognizing Gen-AI’s limitations, including potential inaccuracies and biases, and fostering critical evaluation of AI-generated information.

EDU.3. How can cyber–human interaction be structured in education?

Researchers should focus on establishing a sound educational reason for using AI, focusing on supporting learning rather than replacing human effort, and ensuring AI use supports creative learning and human agency. We could set clear guidelines for appropriate AI use, emphasizing that AI should expand upon, not replace, students’ own ideas and voices. Also, we should go beyond simple question and answer prompting of AI tools to structured prompts that engage humans and AI in dialogical interactions, using question-based feedback to enhance creative thinking rather than providing direct answers.

EDU.4. How can cyber-creativity support and not undermine teachers?

We should explore strategies and co-creation models that empower teachers to adopt AI as a supportive creative collaborator rather than a replacement, building trust and confidence, and evaluate how integrating AI tools into teaching practices enhances teachers’ cyber-creativity competencies and improves academic outcomes. We could research the development of integrated career guidance systems that combine algorithmic insights with human mentoring to help students and families navigate an evolving job market. We can investigate interventions and professional development programs that redefine the teacher’s role as a mentor and creative facilitator in AI-rich classrooms, while supporting teachers’ mental well-being.

EDU.5. How to introduce ethical values in the cyber-creative education cycle?

We should develop and test culturally sensitive, adaptable AI-powered educational tools that ensure equitable access and personalized learning experiences for all student populations, particularly the most vulnerable. We could design hybrid pedagogical models that balance AI assistance with human-led instruction to actively foster critical thinking and creativity and evaluate their impact on students’ mental health. Studies could explore how governments, non-profits, and private sectors can collaborate to provide affordable or subsidized AI-driven resources in underserved areas, thus minimizing the Matthew Effect and promoting inclusive education (see Pedró et al. 2019). Research could focus on ensuring that AI algorithms are free from biases (also in creativity assessment) and support diverse learning needs to create a more ethical and responsible educational environment for the development of positive creativity.

EDU.6. What is the potential of cyber-creativity in supporting life-long learning?

Given the pace at which knowledge evolves, life-long learning appears today to be a must more than an option. Cyber-creativity could be a crucial element in this process. We should develop and evaluate adaptive AI-driven personalized learning systems that continuously adjust to individual learners’ needs and desires, ensuring ongoing professional development and creative skill acquisition, while studying the socio-emotional impacts of AI integration on motivation and educational equity.

## 14. Cyber-Creativity: Ethical Aspects

a. **State-of-the-art**

Recent developments in AI, especially generative systems, have raised significant ethical concerns in the realm of creative production (Floridi 2023, 2024). Existential risks, i.e., the possibility that humanity might be terminated due to the advent of AI, are not excluded even by AI experts^1^. On a more specific level, creativity researchers are urged to consider the ethical implications of cyber-creativity, as it is known that innovations celebrated for their brilliance at the outset later reveal unforeseen societal costs (Runco and Albert 2010; Sawyer 2005, 2010).

Protecting creators is a priority; ethical frameworks now call for consent mechanisms ensuring that artists’ works are used only with explicit permission and proper attribution (Bianco et al. 2023; Foscarin et al. 2020). Ownership and authorship issues are under scrutiny, with emerging legal and transparency guidelines aimed at preventing the unapproved appropriation of creative styles (Rotolo 2025). Transparency in AI, supported by open-source initiatives and detailed explainability reports (Ali et al. 2023; Ben-Tal et al. 2020; Louie et al. 2020), is essential to build trust among stakeholders and safeguard artistic integrity (Schelble et al. 2024).

Bias and cultural representation remain pressing issues. The predominance of Western-centric, WEIRD datasets (Henrich 2020) has led to outputs that often misrepresent or exclude non-Western art forms, risking cultural homogenization (Borenstein and Howard 2021; Tubaro and Casilli 2019). This can lead to AI misinterpreting cultural nuances, leading to biased output. For instance, if one asked an image-producing AI model to generate a Warli painting—tribal art unique to the state of Maharashtra in India—it may produce inauthentic output if it is never trained on representative data. Biased models could therefore restrict the ethical usage of AI to a few specific cultures and nations. Researchers should advocate for diversified training data and advanced debiasing techniques to ensure that AI systems respect and reflect global cultural diversity (Ferrara 2023).

Moreover, ethical research in cyber-creativity increasingly emphasizes the need for regulatory frameworks that promote benevolent, fair, and sustainable AI outputs. This includes developing monitoring systems to prevent harmful content, ensuring that AI-generated creative works align with human values, and even fostering technomoral virtues like honesty, empathy, and transparency in AI design (Vallor 2016). Collectively, these state-of-the-art efforts aim to ensure that as AI continues to evolve, it does so in a manner that supports creative expression while protecting the rights, diversity, and well-being of all stakeholders.

b. **Research challenges for ethics in cyber-creativity**

ETHICS.MAIN: *Understand ethical implications of cyber-creativity in terms of benevolence, fairness, equity, inclusivity, accessibility, sustainability, trust, explainability, and alignment with human values.*

Researchers should investigate the ethical implications of cyber-creativity in terms of benevolence, fairness, equity, inclusivity, accessibility, sustainability, trust, explainability, and alignment with human values. This requires a multidimensional framework that evaluates both the procedural and outcome-based ethical dimensions while capturing the interplay between AI systems and human creative practices. Researchers shall distinguish the technical efficiencies of AI outputs from the moral and humanistic considerations inherent in genuine creative endeavors, addressing evolving norms around bias, transparency, and accountability in hybrid processes. Ultimately, the goal is to develop rigorous, context-sensitive ethical evaluation methods that ensure cyber-creativity upholds human values and fosters a fair, inclusive, and sustainable creative ecosystem. This main research challenge contains a large set of more specific questions, among which are the following six.

ETHICS.1. How can the identity of artists and content creators be protected?

We should investigate and refine consent mechanisms and attribution protocols to ensure that AI-generated art respects the unique styles and rights of artists and content creators. Research should focus on developing legal and technological safeguards that protect identity and maintain artistic integrity while allowing transparent collaboration with AI (Bianco et al. 2023; Foscarin et al. 2020).

ETHICS.2. How can bias be mitigated in cyber-creative practices?

We should address the risk of cultural and demographic biases by designing diversified training datasets and debiasing techniques. We should aim to improve AI’s cultural sensitivity so that creative outputs reflect global diversity and avoid reinforcing stereotypes (Borenstein and Howard 2021; Tubaro and Casilli 2019). AI may propagate biases to the degree that it has been trained on information or data that are reflective of prejudice.

ETHICS.3. How can transparency and accountability be fostered?

We should explore methods to enhance the transparency of AI systems, such as explainability reports that detail data sources and algorithmic processes. These approaches can build trust and ensure that AI’s role in creative production is clear, enabling better accountability for ethical outcomes (Ben-Tal et al. 2020; Louie et al. 2020).

ETHICS.4. What regulatory framework is both sufficient and acceptable for AI?

We should research possible governance and regulatory frameworks and monitoring systems at a global level (Roberts et al. 2024) to prevent the generation of distrustful, harmful, or unethical cyber-creative systems, promoting trustworthiness, benevolence, and sustainability. This includes developing strategies that ensure AI outputs contribute positively to society (Ali et al. 2023).

ETHICS.5. How can technomoral virtues be integrated into cyber-creative processes?

We should investigate how virtues such as honesty, empathy, and humility can be embedded in AI development for cyber-creativity. This line of research could provide guidelines for ethically aligning AI with human values, ensuring that technological advancement supports rather than detracts from creative integrity (Vallor 2016).

ETHICS.6. How can we balance equity and accessibility to AI in cyber-creativity?

We should examine how the widespread use of AI in creative industries may impact equity. At the same time, research should address whether making creativity more accessible through AI might inadvertently reduce the unique advantages that underrepresented groups derive from creative expression (OECD 2021; Pew Research Center 2023).

ETHICS.7. How can ethical oversight and human control be established?

We should design real-time governance models and ethical control interfaces that embed human judgment into AI-generative processes. We could help ensure that moral and ethical standards are maintained, even as systems become more autonomous.

## 15. The Dark Side of Cyber-Creativity

a. **State-of-the-art**

Cyber-creativity, while celebrated for its innovative potential, also harbors a dark side when harnessed with malevolent intent. At its core, creativity is neutral, defined by originality and effectiveness, but when directed toward harm, it becomes what scholars call “malevolent creativity” (Baas et al. 2019; Cropley et al. 2010). Historically, humans have demonstrated the capacity for malevolent creativity, using their ingenuity and moral disengagement to devise new methods of deception, violence, and fraud (Cheng et al. 2021; Harris and Reiter-Palmon 2015; Kapoor and Kaufman 2022; Segatta et al. 2025).

In the AI era, three conditions may foster malevolent outcomes. First, human agents can independently produce harmful creative content. Second, collaborative efforts between humans and AI can amplify destructive capabilities, evident in the rise of fake content, persuasive disinformation, and criminal use of generative AI (e.g., deepfakes, automated phishing) (Rafner et al. 2023). Third, questions arise about whether AI, when trained to produce harmful outcomes, might autonomously generate malevolent content. While AI lacks inherent intentionality, its outputs can be directed by human input, blurring the lines between human and machine responsibility.

Furthermore, the integration of AI in creative fields magnifies ethical dilemmas, such as the potential spread of biased content and the risk of undermining societal trust. AI systems often rely on training datasets that are Western-centric, risking cultural homogenization and misrepresentation of diverse artistic traditions (Tubaro and Casilli 2019). As AI becomes more embedded in creative production, safeguarding against its misuse, whether through fraudulent forgery, deepfake identity theft, or even existential threats, has become increasingly urgent. Researchers are now tasked with not only understanding these dark dimensions but also devising countermeasures to mitigate them, ensuring that the benefits of cyber-creativity do not come at the expense of ethical integrity and human well-being.

b. **Research challenges to combat the dark side of cyber-creativity**

DARK.MAIN: *Develop safeguards against malevolent, biased, fraudulent, misaligned, and unfair use of cyber-creativity as well as against the existential risk for humanity.*

Researchers should develop safeguards against the malevolent, biased, fraudulent, misaligned, and unfair use of cyber-creativity as well as against the existential risks it may pose to humanity. This requires a multidimensional framework that assesses not only the technical capabilities of AI-driven creative outputs but also their potential to deceive, manipulate, and inflict harm. Researchers must distinguish between benign innovations and those deliberately engineered for destructive purposes while integrating ethical, legal, and technical countermeasures. Ultimately, the goal is to establish rigorous, context-sensitive protective strategies that ensure cyber-creativity promotes societal well-being and cultural integrity rather than undermine them. This main research challenge contains a large set of more specific questions, among which are the following six.

DARK.1. How should the malevolent use of cyber-creativity be contrasted?

The dark side of cyber-creativity has the potential to cause very significant damage to society at large, affecting multiple domains and creating a climate of fear and mistrust in the population. Researchers should study forms of governmental control, law-enforcement, and punishment against cyber-creative criminals.

DARK.2. How can infinite recursive cyber-creative generation be counteracted?

We should implement monitoring and limiting frameworks to detect and restrict unbounded cyber-creative content generation, which would have the potential to destroy any creative domain. Researchers could focus on curbing the proliferation of harmful or disruptive outputs while preserving creative freedom.

DARK.3. How could deepfake and identity fraud be counteracted?

We should face the challenge of developing real-time, explainable systems that can reliably detect and attribute AI-generated deepfakes and identity fraud, which are dystopian forms of cyber-creativity. As researchers, we should aim to provide forensic traceability and robust protection of personal identity, keeping pace with the rapid evolution of Gen-AI technologies.

DARK.4. How can automated phishing and social engineering be mitigated?

Adaptive machine learning models that analyze linguistic, contextual, and behavioral patterns to identify cyber-creative phishing attempts should be developed. Research could focus on integrating these models into broader security frameworks, enhancing digital safety for all users.

DARK.5. How can we counteract disinformation/propaganda enabled by cyber-creativity?

We should establish verification frameworks that combine network analysis, content evaluation, and human fact-checking. Researchers should explore ways to flag and neutralize AI-driven disinformation while still upholding free speech and privacy.

DARK.6. How can forgery and fraud be prevented in cyber-creative industries?

It would be important to develop advanced digital forensic methods—such as blockchain authentication and watermarking—to verify the provenance of cyber-creative works. Researchers should ensure these tools can reliably distinguish genuine art from AI-generated counterfeits.

## 16. Conclusions

The main research challenges and specific research questions in the decalogue are summarized in Table 3. This article is intended to be an open door towards future research activities, and as such, it cannot be concluded in a traditional way. At any rate, several comments are in order. Even though we found it useful to structure our development over the ten dimensions of the decalogue of challenges, it should be clear that no classification is complete or perfect. The decalogue is only a useful and systematized frame of reference. It is definitely possible to find overlapping elements between different dimensions, as well as topics that are horizontal to all dimensions.

The hope for this work is that it can stimulate a reflection about the multifaceted implications of the emergence of AI on the scene of human creativity, and that this reflection will rapidly give life to action-oriented research projects, the results of which might give a small but significant contribution towards the fulfilment of the most beneficial promises of AI, while avoiding the undeniable dangers. Shaping the trajectories of cyber-creativity evolution will be a collective effort, and each researcher in this field shall have a role, albeit perhaps a small one. Knowledge about cyber-creativity should not remain confined within the walls of academia but should rather be disseminated to the general public and policy makers, in order to increase awareness and alignment and provide positive contribution to the evolution towards the Post-Information Society.

## Figures and Tables

**Table 1:** Superposition of the decalogue, 4Ps, 5As, and 7Cs frameworks.

Decalogue	4Ps (Rhodes 1961)	5As (Glăveanu 2013)	7Cs (T. Lubart 2017)
1. Theory			
2. Sociocultural	Press	Audience + Affordances	Context
3. Process	Process	Action	Creating
4. Agent	Person	Actor	Creator
5. Team			Collaboration
6. Products	Product	Artifact	Creation + Consumption
7. Domains			
8. Education			Curricula
9. Ethics			
10. Dark side			

**Table 2:** DUCP layers of complexity and creativity forms (adapted from Corazza 2019).

Layer of Complexity	DUCP Dimension	Creativity Form	Characteristics
Material layer	Material Creative Process	Wide-sense	Emergent and Energy-Driven
Biological layer	Biological Creative Process	Wide-sense	Emergent and Adaptive
Psycho-Social layer	Psycho-Social Creative Process	Strict-sense	Intentional and Autonomous
Artificial layer	AI Creative Process	Wide-sense	Computational

**Table 3:** Summary of main research challenges and research questions in the decalogue.

Cyber-Creativity		
**1. Theoretical Framework**	Th.1	What defines cyber-creativity as a distinct field of study?
Develop an interdisciplinary theoretical framework that integrates all forms of creativity, in-cluding human and artificial ones	Th.2	How will cyber-creativity transform established epistemological frameworks?
	Th.3	What is the role of cyber-creativity in a cosmological perspective?
	Th.4	What frameworks are needed for the study of cyber-creativity?
	Th.5	What methodologies are most effective for researching cyber-creativity?
	Th.6	How is creativity evolving in the artificial layer of complexity?
**2. Sociocultural Perspectives**	So.1	How will creative work be distributed in the socio-cultural milieu?
Understand how cyber-creativity shapes and is shaped by the socio-cultural fabric of the post-information society	So.2	How will AI be integrated across global social-technological systems?
	So.3	How can sociocultural feedback loops and system dynamics be analyzed?
	So.4	How should cyber-creativity research act as a social feedback mechanism?
	So.5	What could foresight methodologies bring in envisioning possible futures for cyber-creativity?
	So.6	How might cultural and epistemological assumptions about problems be challenged?
**3. Cyber-Creative Process**	Pr.1	How to optimize cyber-human collaboration in the Drive state?
Model the interaction of human and artificial agents in every phase of the cyber-creative process	Pr.2	How to optimize cyber-human collaboration in Information gathering?
	Pr.3	How could cyber-human collaboration be optimized for Novelty generation?
	Pr.4	How could human and artificial agents collaborate in Creativity estimation?
	Pr.5	How efficient could cyber-human Implementation be?
**4. Cyber-Creative Agent**	Ag.1	How will creative work be distributed in the socio-cultural milieu?
Understand how cyber-creativity shapes and is shaped by the socio-cultural fabric of the post-information society	Ag.2	How will AI be integrated across global social-technological systems?
	Ag.3	How can sociocultural feedback loops and system dynamics be analyzed?
	Ag.4	How should cyber-creativity research act as a social feedback mechanism?
	Ag.5	What could foresight methodologies bring in envisioning possible futures for cyber-creativity?
	Ag.6	How might cultural and epistemological assumptions about problems be challenged?
**5. Cyber-Creative Team**	Te.1	How can we facilitate cognition beyond idea generation in human-AI teaming?
Study the psychological and sociocultural characteristics of a cyber-human team engaged in a cyber-creative process	Te.2	How effective are AI tools in enhancing teams’ cyber-creativity?
	Te.3	How does AI reshape collective intelligence in cyber-creative teams?
	Te.4	What multifaceted roles can AI assume within cyber-creative teams?
	Te.5	What is the potential for dynamic role switching in cyber-creative teams?
	Te.6	How does the trust building process work in cyber-creative teams?
	Te.7	How can we define effective leadership in a cyber-creative team?
**6. Cyber-Creative Products**	Pd.1	How can we intend and determine authenticity for cyber-creative products?
Characterize cyber-creative products in terms of meaningfulness, originality, effectiveness, au-thenticity, ownership, authorship, acceptance, and dynamic potential.	Pd.2	How do perceptual biases influence the evaluation of cyber-creative products?
	Pd.3	How can automated creativity scoring methods be enhanced?
	Pd.4	How can we differentiate levels of creativity in cyber-creative outputs?
	Pd.5	How can machine perception capabilities be developed?
	Pd.6	How can we ensure product diversity and mitigate homogenization?
	Pd.7	How does trust in AI affect cyber-creative product perception?
**7. Cyber-Creative Domains**	Do.1	How does cyber-creativity manifest itself in specific domains?
Explore the specific and diversified impact of cyber-creativity on different domains.	Do.2	What frameworks enable effective cyber-creativity in specific domains?
	Do.3	How will cyber-creativity evolve in professional environments?
	Do.4	How to challenge professional domain boundaries in the age of cyber-creativity?
**8. Cyber-Creative Education**	Ed.1	How can AI augment the creative learning experience?
Design pedagogies for cyber-creative teaching, cyber-creative learning, and cyber-creative development.	Ed.2	How can AI support the development of critical thinking?
	Ed.3	How can cyber-human interaction be structured in education?
	Ed.4	How can cyber-creativity support and not undermine teachers?
	Ed.5	How to introduce ethical values in the cyber-creative education cycle?
	Ed.6	What is the potential of cyber-creativity in supporting life-long learning?
**9. Cyber-Creativity: Ethical Aspects**	Et.1	How can the identity of artists and content creators be protected?
Understand ethical implications of cyber-creativity in terms of benevolence, fairness, equity, inclusivity, accessibility, sustainability, trust, explainability, and alignment with human values.	Et.2	How can bias be mitigated in cyber-creative practices?
	Et.3	How can transparency and accountability be fostered?
	Et.4	What regulatory framework is both sufficient and acceptable for AI?
	Et.5	How can technomoral virtues be integrated into cyber-creative processes?
	Et.6	How can we balance equity and accessibility to AI in cyber-creativity?
	Et.7	How can ethical oversight and human control be established?
**10. Dark Side of Cyber-Creativity**	Da.1	How should the malevolent use of cyber-creativity be contrasted?
Develop safeguards against malevolent, biased, fraudulent, misaligned, unfair use of cyber-creativity as well as against the existential risk for humanity.	Da.2	How can infinite recursive cyber-creative generation be counteracted?
	Da.3	How could deepfake and identity fraud be counteracted?
	Da.4	How can automated phishing and social engineering be mitigated?
	Da.5	How can we counteract disinformation/propaganda enabled by cyber-creativity?
	Da.6	How can forgery and fraud be prevented in cyber-creative industries?

## Data Availability

The original contributions presented in this study are included in the article. Further inquiries can be directed to the corresponding author(s).

## Note

1 https://aistatement.com/ accessed on 2 July 2025.
